# Neuro-Bridge-X: A Neuro-Symbolic Vision Transformer with Meta-XAI for Interpretable Leukemia Diagnosis from Peripheral Blood Smears

**DOI:** 10.3390/diagnostics15162040

**Published:** 2025-08-14

**Authors:** Fares Jammal, Mohamed Dahab, Areej Y. Bayahya

**Affiliations:** 1 Department of Computer Science, Faculty of Computing and Information Technology, King Abdulaziz University, Jeddah 22254, Saudi Arabia; 2 Department of Software Engineering, College of Engineering, University of Business and Technology, Jeddah 21361, Saudi Arabia

**Keywords:** Acute Lymphoblastic Leukemia (ALL), neuro-symbolic hybrid model, peripheral blood smear analysis, Meta-XAI and Explainable AI, vision transformer with fuzzy logic reasoning

## Abstract

**Background/Objectives:** Acute Lymphoblastic Leukemia (ALL) poses significant diagnostic challenges due to its ambiguous symptoms and the limitations of conventional methods like bone marrow biopsies and flow cytometry, which are invasive, costly, and time-intensive. **Methods:** This study introduces Neuro-Bridge-X, a novel neuro-symbolic hybrid model designed for automated, explainable ALL diagnosis using peripheral blood smear (PBS) images. Leveraging two comprehensive datasets, ALL Image (3256 images from 89 patients) and C-NMC (15,135 images from 118 patients), the model integrates deep morphological feature extraction, vision transformer-based contextual encoding, fuzzy logic-inspired reasoning, and adaptive explainability. To address class imbalance, advanced data augmentation techniques were applied, ensuring equitable representation across benign and leukemic classes. The proposed framework was evaluated through 5-fold cross-validation and fixed train-test splits, employing Nadam, SGD, and Fractional RAdam optimizers. **Results:** Results demonstrate exceptional performance, with SGD achieving near-perfect accuracy (1.0000 on ALL, 0.9715 on C-NMC) and robust generalization, while Fractional RAdam closely followed (0.9975 on ALL, 0.9656 on C-NMC). Nadam, however, exhibited inconsistent convergence, particularly on C-NMC (0.5002 accuracy). A Meta-XAI controller enhances interpretability by dynamically selecting optimal explanation strategies (Grad-CAM, SHAP, Integrated Gradients, LIME), ensuring clinically relevant insights into model decisions. **Conclusions:** Visualizations confirm that SGD and RAdam models focus on morphologically critical features, such as leukocyte nuclei, while Nadam struggles with spurious attributions. Neuro-Bridge-X offers a scalable, interpretable solution for ALL diagnosis, with potential to enhance clinical workflows and diagnostic precision in oncology.

## 1. Introduction

Acute Lymphoblastic Leukemia (ALL) is a haematological malignancy characterized by the overproduction of immature white blood cells in the bone marrow, leading to impaired immune function and potentially life-threatening complications. As the most common childhood cancer, ALL makes up about 25% of all paediatric cancer cases [1]. Traditionally, doctors have used invasive methods such as bone marrow biopsies and flow cytometry to diagnose ALL. These methods are painful, expensive, take a lot of time, and need expert knowledge [2]. These problems show the importance of developing non-invasive, effective, and precise diagnostic tools, especially in places with limited resources and less access to modern medical facilities.

Artificial intelligence (AI), especially deep learning, has become a useful tool for many diagnostic problems in recent years. When it comes to identifying and classifying leukemia cells in medical images like peripheral blood smear (PBS) images, deep learning models have shown impressive accuracy [3,4,5]. However, a major challenge in using these models in clinical settings is their “black-box” nature. In crucial medical situations where understanding is essential for clinician acceptance and patient safety, the unclear decision-making process of these models weakens trust and limits their use [6,7].

Explainable AI (XAI) approaches aim to improve the transparency and understanding of AI models [8]. They have gained attention as a way to bridge this gap. To clarify how deep learning models make decisions, XAI techniques like Grad-CAM, SHAP, Integrated Gradients, and LIME have been used in medical imaging. These methods build trust and make it easier to integrate AI into clinical workflows by showing physicians which features of an image influence a model’s predictions [9].

This work presents a new neuro-symbolic hybrid model named Neuro-Bridge-X. It is built to automatically and clearly diagnose acute lymphoblastic leukemia (ALL) using PBS images. Neuro-Bridge-X combines the benefits of vision transformers for contextual encoding, convolutional neural networks (CNNs) for feature extraction, and fuzzy logic-inspired reasoning for decision-making. It merges deep learning with symbolic reasoning. The model uses an adaptive Meta-XAI controller, which picks the best explanation method for each prediction. This hybrid strategy offers both high diagnostic accuracy and understandable explanations. The model performs exceptionally well and generalizes strongly across various optimizers and validation settings when tested on two large datasets, ALL Image and C-NMC.

Although such model agnostic methods as Grad-CAM and SHAP have made a step forward to make deep learning models interpretable, explaining the reasons why one model or an input feature matters more than another, they do not present a structure or a rule-based explanation, which would be comprehensible according to the clinical reasoning. Within areas of diagnosis, including hematology, clinicians have come to depend on logical models that tend to be in the form of IF-THEN sentences to make judgment and convey their conclusion [10]. The visual saliency maps or feature importance scores in themselves do not suffice the demand of cohesive, sequential explanations that can be modified to meet standardized diagnostic criteria. Consequently, even though such XAI methods are convenient to apply in research settings, they are not fully suitable to the requirements of clinical integration that require a high level of transparency, traceability, and reasonable justification. In an effort to fill such interpretability gap, Neuro-Bridge-X develops a neuro-symbolic architecture, which puts symbolic reasoning within the diagnosis process itself. Uniting the possibilities of deep learning in pattern recognition and fuzzy-based rule logic, it generates explanations that, in addition to indicating a relevant feature, express them as statements that are less as sophisticated as they are clinically interpretable [11,12]. Such hybridization increases the levels of both trust and utility, which makes the AI predictions more viable and deployable in the professional context of medicine.

Disease diagnosis, especially in oncology, has changed because of deep learning in medical image analysis [13,14]. Deep learning algorithms have shown great effectiveness in recognizing and classifying leukemia cells in peripheral blood smear images [15]. These images help in diagnosing acute lymphoblastic leukemia (ALL). For instance, Saeed et al. (2022) created a deep learning method that used EfficientNetV2S and EfficientNetB3 architectures. This method accurately distinguished between normal and blast cells in microscopic blood smear images, achieving an impressive accuracy of 99.99% [3]. Shafique and Tehsin (2024) introduced an attention-based deep learning model for ALL classification. This model reached high accuracy and robustness across different datasets [4]. Elsayed et al. (2023) thoroughly examined deep learning algorithms for diagnosing ALL with bone marrow images. Their findings showed the strong potential of these methods to improve diagnostic accuracy [5]. Cheng et al. (2024) looked into deep learning’s role in flow cytometry for diagnosing acute leukemia. They reported a sensitivity of 98.2% for B-lymphoblastic leukemia (B-ALL) and 94.6% for acute myeloid leukemia (AML) [6]. Talaat et al. (2023) explored machine learning techniques for leukemia identification using the C-NMC Leukemia dataset. Their research highlights how AI can enhance diagnostic precision [7].

Despite these advancements, a major challenge in using deep learning models in clinical settings is their interpretability. Several methods have been developed to improve the visibility and reliability of AI models. Explainable AI (XAI) has emerged as an important field focused on this issue. XAI techniques have been applied in medical imaging to clarify model predictions. Honjo et al. (2022) highlighted how XAI methods can improve clinician trust and support decision-making [8]. Ribeiro et al. (2016) introduced LIME, a model-agnostic XAI technique that explains predictions by locally approximating the model’s behaviour [10]. Lundberg and Lee (2017) created SHAP, which uses game theory to assign priority ratings to features, providing a framework to evaluate complex models [16]. Selvaraju et al. (2017) developed Grad-CAM, a technique that identifies important areas in images that influence a model’s decision [17]. Integrated Gradients, introduced by Sundararajan et al. (2017), calculates feature attributions by integrating gradients along a path from a baseline to the input. These techniques have been essential in improving the interpretability of deep learning models in medical contexts [18].

K. Pervez et al. used transfer learning with LIME to diagnose and predict ALL in leukemia diagnosis. They achieved an accuracy of 98.38% and provided justifications for the model predictions [19]. Honjo et al. (2022) explored saliency-based XAI techniques and showed their value in medical imaging applications [8]. In their systematic review of XAI in medical image analysis, Van der Velden et al. (2022) examined more than 200 publications and emphasized the importance of transparency in crucial decision-making [20]. Hou et al. (2024) carried out a thorough study on self-explainable AI for medical image processing and detailed methods and assessment criteria [21].

Neuro-symbolic AI combines neural networks with symbolic reasoning. It has become popular for its strong performance and ability to explain results [22,23,24]. This approach enables models to reason about structured data and provide explanations by merging the logical reasoning of symbolic systems with the pattern recognition strengths of deep learning. Cai et al. provided an overview of neural-symbolic computing, highlighting its potential to overcome the shortcomings of traditional deep learning models [25]. Hadabi et al. discussed the importance of neuro-symbolic techniques in creating reliable and understandable AI systems during their exploration of the third wave of AI [26]. Compared to traditional deep learning models, neuro-symbolic AI has shown improved performance and clarity in medical imaging tasks such as image segmentation and disease detection. However, its application in diagnosing ALL is still uncharted, which leaves opportunities for further exploration.

El Alaoui et al. emphasized the role of deep learning in improving diagnostic results in their review of AI methods for leukemia detection [15]. Recent research has also examined AI applications in similar blood disorders. A 2024 study published in ScienceDaily reported that AI can identify genetic traits from bone marrow smears, which removes the need for expensive genetic testing [27]. Cheng et al. (2024) achieved high predictive accuracy by using machine learning to predict blood parameters in children with acute leukemia [28]. While these studies show the increasing importance of AI in hematology, the use of neuro-symbolic techniques is still not present [28].

The following Table 1 summarizes key studies on deep learning and XAI for ALL diagnosis and medical imaging from 2022 and later.

By introducing Neuro-Bridge-X, a neuro-symbolic hybrid model made for ALL diagnosis, our work builds on current advancements. Neuro-Bridge-X aims to fix the weaknesses of existing AI-based diagnostic tools by using deep learning for feature extraction and symbolic reasoning for decision-making. This approach should provide high accuracy and clear explanations.

By exploiting peripheral blood smear (PBS) images this research work introduces a new Neuro-Bridge-X hybrid neuro-symbolic model of automatic and transparent diagnosis of acute lymphoblastic leukemia (ALL). The primary contributions of this work as follows:

1. Novel Neuro-Symbolic Architecture: Neuro-Bridge-X integrates morphological feature extraction CNN, contextual encoding visual transformer and reasoning under the influences of fuzzy logic to hybridize Deep learning and formal knowledge representation. This mixed method allows the model to come up with sensible choices and high accuracy in diagnostic.
2. Adaptive Explainability: With the given model, a Meta-XAI controller dynamically picks between different explanation approaches such as Grad-CAM, SHAP, Integrated Gradient, and LIME depending on the data fed into the model. This personalization makes the explanations pertinent to every prediction, which makes them more transparent and trustworthy.
3. Exceptional Performance on Many Datasets: Neuro-Bridge-X has significant accurate/generalized results under each two distinct datasets, namely, C-NMC (15,135 images taken on 118 patients) and ALL Image (3256 images of 89 patients). It performs good generalization through validation environments and almost estimated at accuracy (1.0000: ALL, 0.9715: C-NMC) by tuning SGD and Fractional RAdam-based optimizers.
4. Class Imbalance Mitigation: In order to resolve the imbalance in the classes of both datasets and represent the benign and leukemic classes in a balanced way, a sophisticated data augmentation technique was also used in order to achieve a good representation of the classes. This makes the model more generalizable to populations of other patients.
5. Detailed Inspection: The model has been evaluated deeply through five-fold cross-validation and fixed train-test split with three optimizers (Nadam, SGD, and Fractional RAdam). It was found that SGD and Fractional RAdam tend to out-perform Nadam, with SGD optimal accuracy on both datasets being 100%, and Fractional RAdam again second.
6. Future Potential and Clinical Relevance: The configurable, interpretable ALL identification system offered through Neuro-Bridge-X would augment clinical cancer diagnostic performance and procedure. Its capability of giving pertinent explanations and valid forecasts is an important resource to a researcher and a physician.

These contributions fill major gaps in existing diagnostic tools based on AI in the ALL scenario and advance the development of neuro-symbolic AI in medical imaging through the ability to provide a combination of performance and interpretability.

## 2. Materials and Methods

### 2.1. Dataset Description

Both the ALL and C-NMC datasets consist of peripheral blood smear (PBS) images captured using a Zeiss microscope (Oberkochen, Germany) equipped with a 100× magnification lens. Images were acquired under standardized laboratory conditions and saved in JPG format. Prior to modeling, all images were uniformly resized and normalized to reduce variability from lighting and staining inconsistencies. These consistent preprocessing procedures ensure that diagnostic features are preserved while facilitating model generalization across datasets.

Both the ALL and C-NMC datasets reflect a clinically diverse and demographically representative patient population. The ALL dataset includes PBS images from 89 patients ranging in age from 2 to 14 years, with a near-equal gender distribution (46% female, 54% male). The samples span multiple disease stages, including early pre-B, pre-B, and pro-B subtypes, which represent the clinical spectrum of acute lymphoblastic leukemia presentation in pediatric patients. Similarly, the C-NMC dataset comprises 118 patients, also primarily pediatric, with images annotated by expert oncologists across varying stages of leukemia progression. The dataset includes balanced classes of leukemic blasts and normal hematopoietic cells, sourced under real clinical protocols with natural variation in staining intensity and slide preparation. This demographic and clinical heterogeneity closely mirrors real-world conditions, strengthening the model’s relevance and reducing the risk of dataset-induced bias. Such diversity ensures that Neuro-Bridge-X is trained and evaluated on data that truly reflects the variability encountered in everyday medical diagnostics.

#### 2.1.1. Acute Lymphoblastic Leukemia (ALL) Dataset [29]

**Figure 1:**
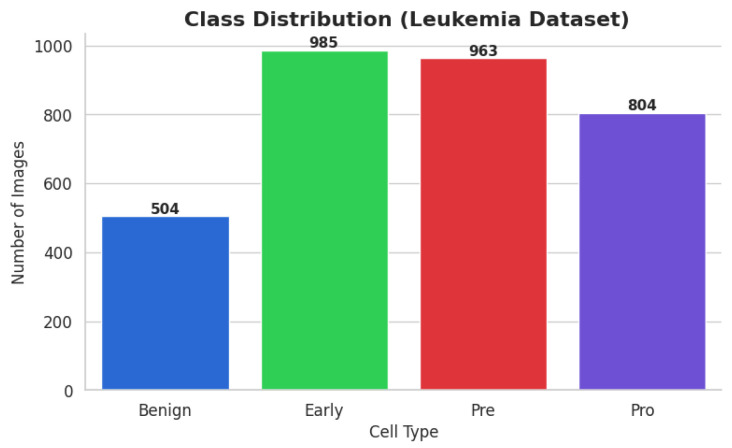
Image count per class ALL dataset.

**Figure 2:**
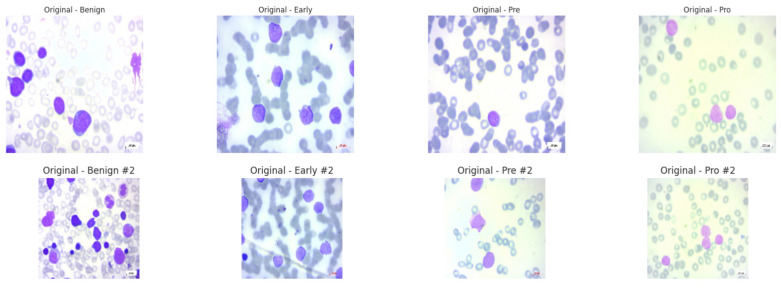
Sample images from ALL dataset at random. Different colors primarily reflect the maturity and density of the cells.

The ALL dataset contains 3256 PBS images from 89 patients, collected and curated at Taleqani Hospital, Tehran. It is annotated into two primary classes: benign (504 images) and malignant (2752 images). The malignant category is further divided into three clinically relevant subtypes Pro-B (804), Pre-B (963), and Early Pre-B (985), which capture the morphological diversity of leukemic cells. Ground truth labels were confirmed via flow cytometry, enhancing the reliability of the annotations. Organized by class in separate folders, the dataset supports structured experimentation. With data sourced from 25 benign and 64 ALL-positive patients, this dataset offers a balanced and clinically validated foundation for developing AI-assisted diagnostic tools. A distribution chart and sample images are shown in Figure 1 and Figure 2.

#### 2.1.2. C-NMC Leukemia Dataset [30]

**Figure 3:**
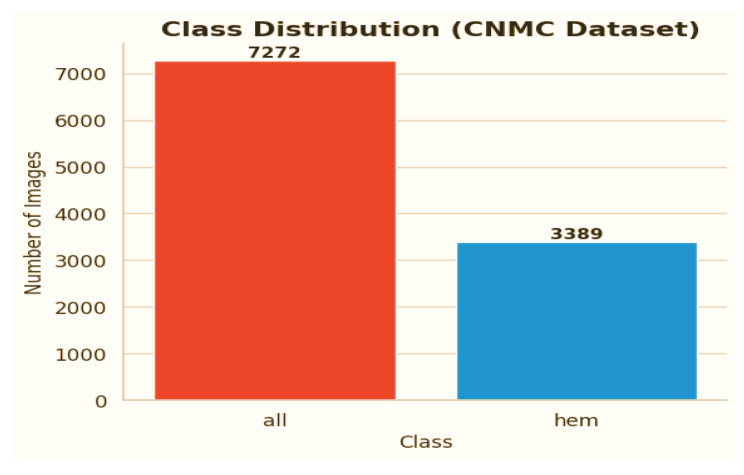
Image count per class CNMC dataset.

**Figure 4:**
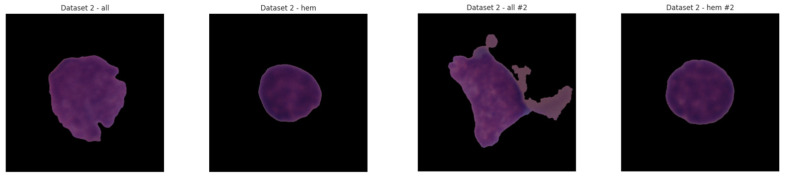
Sample images from CNMC dataset.

C-NMC dataset has 15,135 images of 118 patients, which are divided into leukemia blasts and normal hematopoietic cells. Experienced and skilled oncologists performed labeling in order to make it clinically relevant. The dataset generates little noise of acquisition as compared to the real-world diagnostic settings. It is divided into three k-folds (fold 0, fold 1, fold 2) each of which can be used to train and cross-validate in an iterative manner. Also, a test validation set, coupled with label metadata in CSV format, permits a powerful performance assessment. C-NMC combines the vast coverage of cellular morphology and well-designed design, which makes it an essential benchmarking tool to generalize the capabilities of AI in leukemia classification. Figure 3 and Figure 4 show the class distribution and sample pictures.

### 2.2. Data Preprocessing

An advanced AI model named “Neuro-Bridge-X” was trained on the ALL Image dataset to diagnose Acute Lymphoblastic Leukemia (ALL), and a second dataset, the CNMC Classification dataset, was used to finish this research project. Both types of data are microscopic images of peripheral blood cells and are the main source of information needed to support the development of an automated diagnostic system. Despite its worth, the two datasets show a high degree of class skewness, which could limit the trained model’s ability to generalize predictions across all classes. A comprehensive set of preparation techniques, including class-aware data augmentation, image resizing, and the creation of a special dataset class, were used to partially solve this limitation. These techniques were all intended to improve the quality of the provided data for further model training.

The ALL dataset’s samples are arranged into four groups, each of which stands for a distinct blood cell stage or condition: Pro-leukemia cells, totaling 804 images; pre-leukemia cells, totaling 963 images; early-stage leukemia cells, totaling 985 images; normal cells, totaling 504 images

The study of the statistics makes it evident that there is a notable class disparity. At 985 photos, the “Early” class is the largest class in the sample; at 504 photos, the Benign class is the smallest. This class imbalance could lead to the model overfitting to the more populous classes, thus introducing a bias towards the majority class and decreasing its ability to correctly identify instances from minority classes.

The CNMC Classification Dataset is divided into two classes: Normal cells, comprising 3389 images; Leukemia blasts, comprising 7272 images. Once more, the data analysis makes it evident that this dataset is likewise extremely unbalanced: the “ALL” class is substantially larger than the “HEM” class. This distorted distribution can lead to prediction models that are biased towards the diagnosis of leukemia blasts, which can lead to high false-negative rates of normal cells.

Both datasets exhibit class imbalances, which are frequently addressed and eliminated through the application of class-aware data augmentation approaches. To enable objective model training, a balanced dataset is produced by producing false images for the minority classes and matching their numbers to those of the majority classes.

#### 2.2.1. Data Augmentation for Dataset 1

Increasing the number of images in the Benign, Pre, and Pro classes in the ALL Image Dataset to match the 985 images in the Early class, the standard, in this instance, was the primary goal. Through the augmentation process, the following modifications were performed using Keras 3.11.1 “ImageDataGenerator”. To replicate different orientations, rotation is applied up to 30 degrees. Zoom was modified by up to 20% to replicate varying magnifications; to improve positional diversity, horizontal flipping was used. Brightness Adjustment was performed for illumination variations, ranging from 80% to 120%.

In order to preserve the morphological integrity of the blood cell images and provide sufficient diversity to increase the model’s robustness, these transformations were used. The enhancement yielded the following findings:

- Benign: From 504 to 985 synthetic images, an increase of 481.
- Pre: Grew from 963 to 985 by 22 artificial photographs.
- Pro: From 804 to 985, there were 181 more synthetic photographs.
- Early: Did not need any augmentation, staying constant at 985 photos.

**Figure 5:**
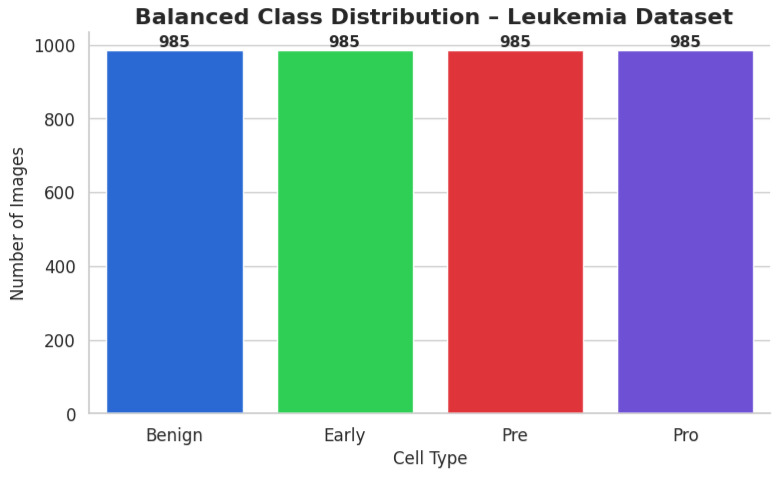
Image count per class after preprocessing ALL dataset.

Each class had its own subfolder within the special directory containing the balanced dataset. To classify the file paths and labels of all the synthetic and real photographs, a comprehensive CSV file was produced. To visually confirm the balancing effort, a bar plot was made to demonstrate that each class now included exactly 985 photographs. After class-aware data augmentation, the class-wise data distribution is shown in Figure 5.

#### 2.2.2. Data Augmentation for Dataset 2

**Figure 6:**
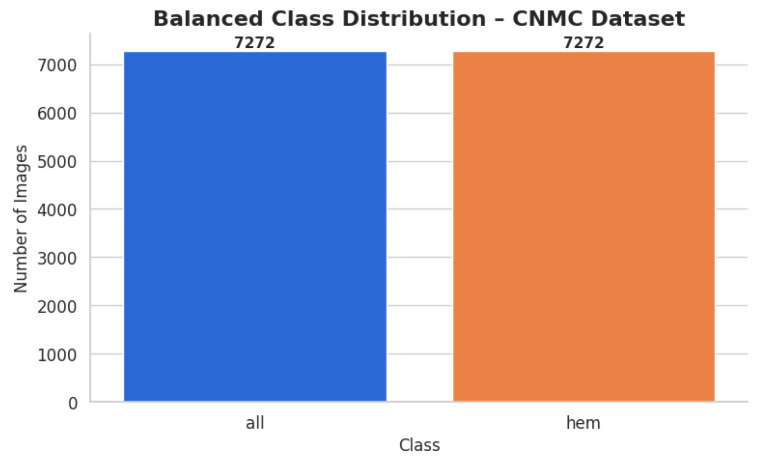
Image count per class after preprocessing—CNMC dataset.

Enhancing the hem class to match the 7272 photos of all classes was the goal for the CNMC Classification Dataset. The same group of augmentation methods were used: Rotation range of 30 degrees and zoom of 20% or more, along with flipping horizontally; adjusting brightness from 80% to 120%. By producing 3883 synthetic photos for the hem class, this process raised the total number of images to 7272 and brought it up to parity with the entire class. The balanced dataset was organized using the subfolders according to the class. A CSV file containing the image paths and labels was created. Then, a bar plot was made to help argue for class size equality. The class-wise data distribution is shown in Figure 6 after class-aware data augmentation.

For the hem class, this procedure produced 3883 synthetic photos, increasing its total to 7272 images and bringing it up to parity with all classes. The class-specific subfolders were used to arrange the balanced dataset. To record the image paths and labels, a CSV file was made. To support the equalization of class sizes, a bar plot was then created. Following class-aware data augmentation, the class-wise data distribution is displayed in Figure 6.

### 2.3. Image Resizing and Dataset Preparation

The sophisticated AI-based model architecture utilized in this experiment was believed to require uniformity in image dimensions in addition to adjusting for class imbalance. Therefore, each image in both datasets was scaled to the standard resolution of 128 by 128 pixels. This dimension was chosen in order to achieve a balance between processing efficiency and preserving sufficient information for feature extraction, both of which are essential for good model training.

A custom dataset class named “LeukemiaDataset” was created using PyTorch 2.7 to speed up the data handling process. This class resizes and converts images into tensor format using the transformations module to ensure a seamless integration of the images into the training pipeline.

To create a model that can learn under supervision, the preprocessing processes indicated above are necessary. By reallocating the representation among the classes, data augmentation overcame the limitation of unbalanced data and countered the dataset’s tendency to be slanted towards majority cases. The controlled variance generated by the use of simulated samples enhanced the model’s capacity to extrapolate across a variety of inputs. Convolutional neural networks were compatible, all the photos were standardized to the same resolution, and an efficient personal data-loading process was used. These procedures were used to create a solid, balanced dataset, which is required to build a model that can correctly detect leukemia. For the ALL dataset, 3940 training photographs were used, while for the CNMC dataset, 14,544 images were used.

### 2.4. Methodology

**Figure 7:**
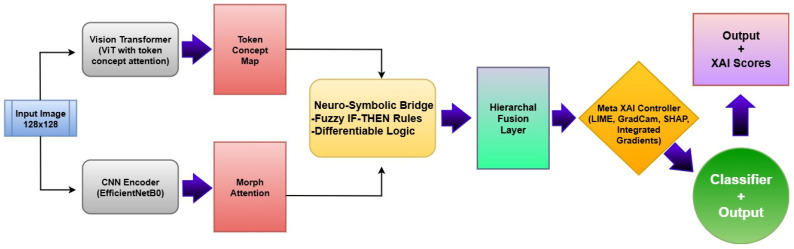
Architectural flowchart of the proposed Neuro-Bridge-X model.

The proposed Neuro-Bridge-X model introduces a novel neuro-symbolic hybrid architecture for automated and explainable diagnosis of leukemia from blood smear images. The framework is engineered by sequentially integrating deep morphological extraction, visual transformer-based conceptual encoding, fuzzy logic-inspired reasoning, and adaptive interpretability control. Each module is described below in detail, along with its mathematical formulation and operational impact on the overall pipeline. The architectural block diagram of Neuro-Bridge-X model is shown in Figure 7.

#### 2.4.1. CNN Encoder with Morphological Attention

The role of the CNN Encoder is to extract low-level and mid-level morphological features that correspond to hematological structures such as leukocyte nuclei, cytoplasmic boundaries, and chromatin textures. These features are pivotal for differentiating leukemia subtypes based on visual pathology.

Let the input image batch be $I\in {\mathbb{R}}^{B\times 3\times H\times W}$ where *B* is the batch size and *H*, *W* are spatial dimensions. The CNN Encoder, denoted $E_{cnn}$, processes this image using the convolutional layers of EfficientNet-B0:

$$F_{cnn}=E_{cnn}(I)\in {\mathbb{R}}^{B\times C\times H^{\prime }\times W^{\prime }}$$

where *C* = 1280, and *H*′, *W*′ are spatial dimensions after down-sampling.

#### 2.4.2. Morphological Attention

To emphasize class-discriminative channels, a channel-wise attention filter is applied. This module uses global average pooling followed by a gating mechanism:

$$\begin{array}{l} z=GAP(F_{cnn})=\frac{1}{H^{\prime }W^{\prime }}\sum_{i=1}^{H^{\prime }}\sum_{j=1}^{W^{\prime }}F_{cnn}\left[:,:,i,j\right]\\ w=\sigma \left(W_{2}.\phi \left(W_{1}.z\right)\right)\in {\mathbb{R}}^{B\times C}\\ F_{att}=F_{cnn}⊙w_{reshaped} \end{array}$$

Here, *ϕ*(⋅) denotes the ReLU activation, and *σ*(⋅) is the Sigmoid function. The attention-modulated feature map F_att_ highlights key channels and suppresses irrelevant background variations.

This module significantly reduces redundancy and emphasizes morphologically salient regions, which improves feature quality for downstream reasoning and reduces overfitting on irrelevant textures.

#### 2.4.3. Vision Transformer (ViT) with Token-Wise Conceptual Attention

While the CNN module encodes spatially local information, the Vision Transformer captures long-range dependencies and contextual semantics by analyzing the entire image through self-attention. It also generates fine-grained patch-level concepts, which are then refined via attention weights.

The image is first decomposed into a sequence of non-overlapping patches of size *P* × *P*, flattened and linearly projected into embedding vectors as follows:

$$X_{p}=PatchEmbed(I)\in {\mathbb{R}}^{B\times N\times D}$$

where $N=\frac{H\times W}{P^{2}}$, and *D* is the embedding dimension.

The positional encoding $P_{pos}\in {\mathbb{R}}^{1\times \left(N+1\right)\times D}$ is added to maintain spatial consistency as follows:

$$T_{0}=[t_{cls}||X_{p}]+P_{pos}$$

Through *L* transformer blocks $\tau_{l}$, self-attention refines token dependencies as follows:

$$T_{L}=\tau_{L}\circ \cdot \cdot \cdot \circ \tau_{1}\left(T_{0}\right)\in {\mathbb{R}}^{B\times (N+1)\times D}$$

#### 2.4.4. Conceptual Attention

Each token is weighted using a learnable attention mechanism:

$$\begin{array}{l} \alpha_{i}=\frac{\exp\left(v^{T}\phi \left(WT_{L,i}\right)\right)}{\sum_{j=1}^{N+1}\exp\left(v^{T}\phi \left(WT_{Lj}\right)\right)}\\ T_{attn}=\sum_{i=1}^{N+1}\alpha_{i}.T_{L,i} \end{array}$$

The conceptual attention mechanism filters transformer tokens, allowing only contextually relevant concepts (e.g., blast cell morphology) to influence the symbolic reasoning phase. This improves model focus on semantically rich patches.

#### 2.4.5. Neuro-Symbolic Bridge (Differentiable Logic Layer)

This layer serves as a symbolic interface that combines CNN morphology with ViT concepts and transforms them into a unified, interpretable reasoning vector. It mimics logical inference rules via differentiable transformations.

The CNN features are pooled globally as follows:

$$f_{cnn}=GAP(F_{att})\in {\mathbb{R}}^{B\times 1280},f_{vit}=T_{att}\in {\mathbb{R}}^{B\times 768}$$

These are concatenated and passed through a two-layer logic module as follows:

$$f_{bridge}=\tanh\left(W_{2}.\phi \left(W_{1}.\left[f_{cnn}||f_{vit}\right]\right)\right)\in {\mathbb{R}}^{B\times 256}$$

This module acts as an inference engine where fuzzy interactions between morphological and conceptual features are captured. It enables the model to simulate IF-THEN reasoning patterns, crucial for biomedical interpretability.

#### 2.4.6. Meta-XAI Controller

##### Training Procedure

The Meta-XAI controller in Neuro-Bridge-X is trained end-to-end alongside the main classification pipeline to optimize both diagnostic accuracy and interpretability. After extracting image features via a CNN encoder with morphological attention and a concept-aware Vision Transformer, these representations are passed through a neuro-symbolic bridge that fuses them into a compact 256-dimensional logic-informed feature vector. This output is simultaneously fed into two parallel modules: the final classifier and the Meta-XAI controller. The controller employs a lightweight neural network to assign dynamic weights across four popular explainability techniques, Grad-CAM, SHAP, Integrated Gradients, and LIME, based on the logic-layer feature context. Training is guided by a composite loss function that integrates cross-entropy classification loss with an auxiliary explanation quality signal, enabling the model to learn to select the most contextually appropriate explanation method for each prediction. This adaptive method enables Neuro-Bridge-X to provide not only accurate conclusions, but also interpretable rationales that are consistent with both the input morphology and the underlying diagnostic logic, increasing its reliability in clinical decision support.

To promote adaptive explainability, this controller selects the most suitable post hoc XAI strategy for each input instance from a predefined set: Grad-CAM, SHAP, Integrated Gradients (IG), and LIME.

$$p_{xai}=soft\max\left(W_{2}^{xai}.\phi \left(W_{1}^{xai}.f_{bridge}\right)\right)\in {\mathbb{R}}^{B\times 4}$$

Each element $p_{k}$ in $p_{xai}$ represents the probability of selecting explanation method *k* for a given sample.

This controller prevents one-size-fits-all explanation policies. Instead, it offers patient-specific interpretability by dynamically adjusting the explanation method best suited to the internal feature configuration.

##### Meta-XAI Controller’s Decision Process

The Meta-XAI controller is a dynamic selection mechanism imbued with context-sensitive weights to various explainability methods such as, Grad-CAM, SHAP, Integrated Gradients, and LIME. Instead of having a fixed explanation technique, the controller takes the output of the fusion of logic-level features trained in the neuro-symbolic bridge and runs a lighter-weight neural selector to produce a weight vector pressed out of the SoftMax over the four XAI alternatives. The weights are not hand-tuned and are learned through end-to-end training, which lets the system adaptively focus on explanation methods that best match the properties of the input sample. To give an example, in cases where suitable distinctive spatial properties are evident, like images with large and grouped blast cells, Grad-CAM may stand a better chance of higher weight of selections because it is particularly robust in visual saliency. On the other hand, SHAP or LIME can be used in more ambiguous cases of a sample having a difficult time predicting pixel-level differences in samples or unclear morphology, with interpretations of varying granularity explained by perturbation changes. It allows Neuro-Bridge-X to provide custom, case-specific explanations that allow most clinicians to increase their trust towards AI, as the method of XAI used will indicate the most diagnostically relevant features of an input image.

#### 2.4.7. Classifier Head

This module maps the fused feature representation to discrete diagnostic categories (e.g., normal, early, pre-leukemia, pro-leukemia).

$$\begin{array}{l} y_{\log its}=W_{2}^{cls}.\phi \left(W_{1}^{cls}.f_{bridge}\right)+b^{cls}\\ y_{pred}=soft\max\left(y_{\log its}\right)\in {\mathbb{R}}^{B\times C} \end{array}$$

The cross-entropy loss is minimized during training as follows:

$$L_{CE}=-\sum_{i=1}^{c}y_{i}\log\left(y_{pred,i}\right)$$

The classifier utilizes a distilled and reasoned representation, resulting in high confidence predictions with semantically grounded decision boundaries.

#### 2.4.8. Full Forward Propagation Summary


$$\begin{array}{l} F_{cnn}\to A_{morph}\to f_{cnn},\\ I\to ViT\to f_{vit},\\ \left[f_{cnn}||f_{vit}\right]\to LogicBridge\to f_{bridge},\\ f_{bridge}\to MetaXAI,f_{bridge}\to Classifier \end{array}$$

- **CNN + Morph Attention**: Enhances structural patterns and suppresses noise.
- **ViT + Token Attention**: Highlights global conceptual cues across the image.
- **Neuro-Symbolic Bridge**: Infers logical consistency across features.
- **Meta-XAI**: Provides personalized explainability for human trust.
- **Classifier**: Delivers final diagnostic inference with reduced entropy.

### 2.5. Experimentation

To rigorously evaluate the performance and generalizability of the proposed Neuro-Bridge-X framework, a series of controlled experiments were conducted on two independent blood smear image datasets. The experimental protocol encompassed both fixed split evaluations and 5-fold cross-validation, utilizing a consistent training pipeline across multiple optimization strategies.

#### 2.5.1. Datasets and Experimental Setup

Two publicly available datasets were used:

1. **Dataset 1 (ALL)**: A curated dataset comprising images labeled across four morphological stages that are benign, early, pre-leukemia, and pro-leukemia.
2. **Dataset 2 (CNMC)**: A clinically annotated collection with images categorized as normal or leukemic, collected under variable imaging conditions.

For both datasets, preprocessing involved resizing all images to a fixed dimension of 128 × 128, followed by normalization. Label encoding was performed using a fixed LabelEncoder to ensure class consistency.

#### 2.5.2. Model Training Protocol

Each experiment was conducted for 10 epochs, using the standard cross-entropy loss function $L_{CE}$, which measures the divergence between the predicted class distribution and the ground-truth labels.

For a given batch *B*, with predictions $y_{pred}\in {\mathbb{R}}^{|B|\times C}$ and ground truth labels $y_{true}\in {\left\{1,\ldots ,C\right\}}^{\left|B\right|}$, the objective is

$$L_{CE}=-\frac{1}{\left|B\right|}\sum_{i=1}^{\left|B\right|}\sum_{c=1}^{C}1_{\left[y_{i}=c\right]}.\log\left(y_{pred,i,c}\right)$$

#### 2.5.3. Optimization Strategies

Three distinct optimizers were evaluated to assess sensitivity to gradient update dynamics:

- **Nadam** (Nesterov-accelerated Adaptive Moment Estimation) with a learning rate of 1 × 10^−3^;
- **SGD** (Stochastic Gradient Descent with Momentum) using η = 0.01, momentum μ = 0.9;
- **RAdam** (Rectified Adam) with fractional decay and a fixed learning rate of 5 × 10^−4^.

These optimizers were supplied via callable wrappers to ensure consistency and reproducibility across all training folds.

#### 2.5.4. Fixed Train-Validation Split Evaluation

For both datasets, the initial phase of experimentation used a predefined static split for training and validation. This setup helps evaluate performance in a controlled setting, where class distributions are preserved. Training was conducted using the following configuration:

- **Batch size**: 32;
- **Loss**: Cross-Entropy;
- **Epochs**: 10;
- **Checkpointing**: Final model weights were saved after the last epoch.

At each epoch, accuracy and loss were computed on both training and validation sets to monitor learning dynamics and overfitting tendencies.

#### 2.5.5. Cross-Validation Protocol

To further strengthen the robustness of the evaluation, the Neuro-Bridge-X model was subjected to 5-fold cross-validation on both datasets. The full training set was divided into five non-overlapping subsets, D_1_, D_2_, …, D_5_, and each fold F_k_ used

$$\begin{array}{l} D_{train}=U_{i\neq k}D_{i}\\ D_{val}=D_{k} \end{array}$$

For every fold:

- The model was re-initialized to prevent information leakage.
- A new optimizer instance was generated to reset learning parameters.
- Training and validation metrics were recorded independently.

The fold-wise results were aggregated as follows:

$$MeanAccuracy_{val}=\frac{1}{5}\sum_{k=1}^{5}Acc_{val}^{\left(k\right)},MeanLoss_{val}=\frac{1}{5}\sum_{k=1}^{5}Loss_{val}^{\left(k\right)}$$

#### 2.5.6. Model Checkpointing and Reproducibility

To ensure result traceability, the model state was saved after each fold. This permits retrospective analysis, ensemble methods, or additional post hoc explainability studies. A fixed random seed was used across all K-Fold shuffling operations to maintain consistent fold distributions.

#### 2.5.7. Experimental Objectives

Each set of experiments was conducted with the following objectives in mind:

1. **Assessing Optimizer Influence**: How do different gradient update rules affect convergence, stability, and generalization?
2. **Evaluating Dataset Generalizability**: Does the model trained on morphologically distinct datasets maintain stable performance?
3. **Validating Architectural Design**: Do the morphological, symbolic, and conceptual components of Neuro-Bridge-X contribute positively under variable training regimes?

This structured experimental design allows comprehensive benchmarking of the Neuro-Bridge-X framework, providing insights into its learning characteristics and generalization performance across hematological imaging scenarios. The summary of experimental coverage is shown in Table 2.

## 3. Results

To evaluate the efficacy and generalizability of the proposed NeuroBridge-X architecture, a comprehensive set of experiments was conducted on two independent leukemia image datasets, ALL (Dataset 1) and CNMC (Dataset 2), using three different optimization strategies: NAdam, SGD, and Fractional RAdam. The results are reported for both 5-fold cross-validation and fixed train-test split settings. Evaluation metrics include accuracy, precision, recall, F1-score, and ROC-AUC (where applicable), offering a multifaceted perspective on the model’s predictive capabilities.

Although data augmentation techniques such as rotation, brightness adjustment, and scaling were employed during training to enhance feature diversity and model robustness, all evaluations were conducted exclusively on real, non-augmented test sets. These held-out samples were not seen during training and were drawn directly from the original clinical datasets, preserving authentic variability in staining, imaging conditions, and cell morphology. Neuro-Bridge-X consistently achieved high accuracy and stability across these unseen test sets, confirming that its predictive performance is not reliant on synthetic patterns. This distinction between augmentation-driven training and real-data evaluation ensures that the model’s performance metrics genuinely reflect its effectiveness in practical clinical settings. Consequently, Neuro-Bridge-X demonstrates strong potential for deployment in real-world diagnostic workflows, where generalization to new and unmodified samples is critical.

### 3.1. Performance on ALL Dataset

#### 3.1.1. Cross-Validation Results

**Figure 8:**
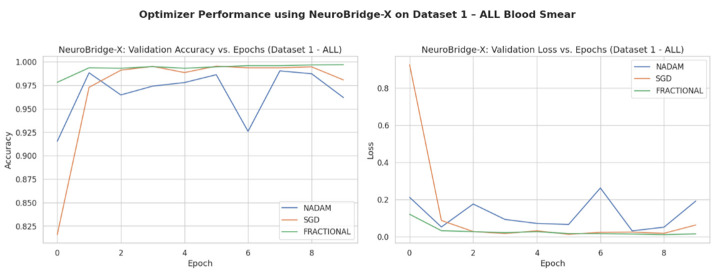
Optimizer wise learning behavior of the proposed model on ALL dataset.

**Figure 9:**
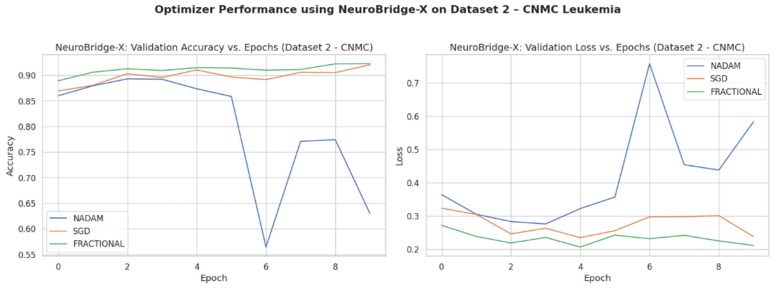
Optimizer wise learning behavior of the proposed model on CNMC dataset.

Across five validation folds, the model demonstrated strong performance across all optimization schemes, with particularly outstanding results under SGD and Fractional RAdam. The learning behavior of the proposed model on ALL dataset and CNMC dataset is shown in Figure 8 and Figure 9, respectively.

- **SGD** achieved a perfect classification across all four leukemia stages (Benign, Early, Pre, Pro), yielding an overall accuracy of 1.0000, with precision, recall, and F1-score all registering at 1.00 for each class. This consistency suggests that the SGD optimizer facilitates stable and accurate gradient convergence across morphologically distinct samples.
- **Fractional RAdam** produced similarly robust outcomes, with a marginal drop in the accuracy score (0.9975). Slight fluctuations in class-wise recall (e.g., Pre = 0.99) accounted for this deviation, yet the macro-averaged and weighted F1-scores remained at unity.
- **NAdam**, while still performing admirably (accuracy = 0.9061), displayed more variability. Particularly, the model achieved near-perfect recall on Early and Pre stages, but its ability to distinguish the Pro stage deteriorated (recall = 0.66), suggesting possible over-smoothing or instability in deeper decision boundaries under this optimizer.

**Figure 10:**
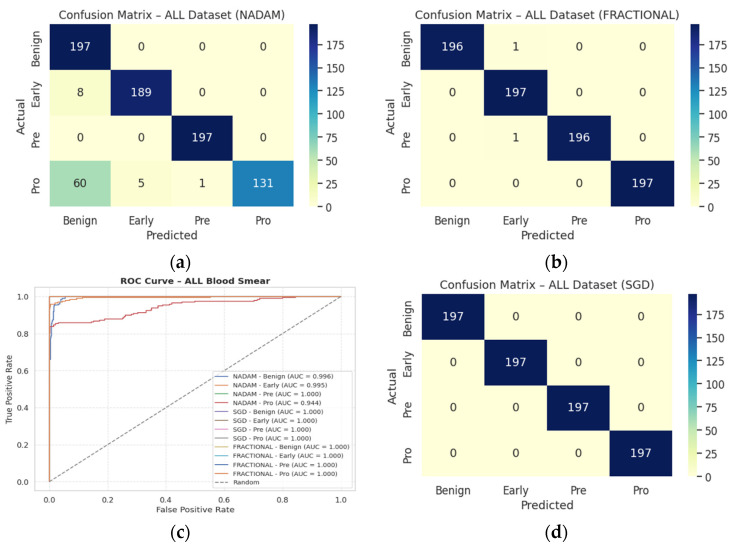
CM under 5-fold cross validation—ALL dataset: (**a**) Nadam, (**b**) Fractional, (**c**) AUC-ROC Curve, (**d**) SGD.

These findings emphasize that SGD and RAdam may be better suited for datasets with nuanced class separability, as they appear less susceptible to performance degradation in minority or visually ambiguous classes. The evaluation metrics obtained during the comprehensive evaluation of the model are summarized in Table 3 followed by the confusion matrices AUC-ROC curve in Figure 10.

#### 3.1.2. Fixed Split Results

**Figure 11:**
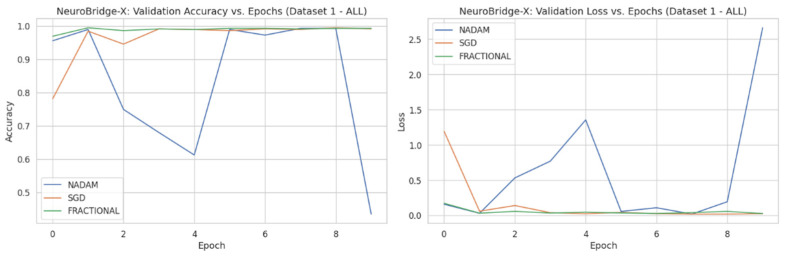
Optimizer wise learning behavior of the proposed model on ALL dataset.

**Figure 12:**
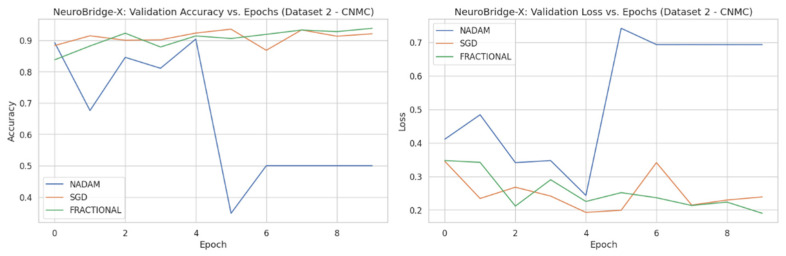
Optimizer wise Learning behavior of the proposed model on CNMC dataset.

The fixed train-test partition reinforced trends observed during cross-validation are as follows:

- **SGD** preserved its dominance, again reaching near-perfect classification across all classes with an accuracy of 0.9983.
- **Fractional RAdam** maintained a comparable performance (accuracy = 0.9932), with only minimal variance in class-wise recall (e.g., Pre = 0.99).
- In stark contrast, **NAdam** exhibited noticeable underperformance (accuracy = 0.4264). While it recalled Early cases accurately (recall = 1.00), it failed to identify the Pro class (recall = 0.00) and severely misclassified Pre cases (recall = 0.06). This suggests a sensitivity to data imbalance or loss curvature that adversely affected convergence. The learning behavior of the proposed model on ALL dataset and CNMC dataset is shown in Figure 11 and Figure 12, respectively.

**Figure 13:**
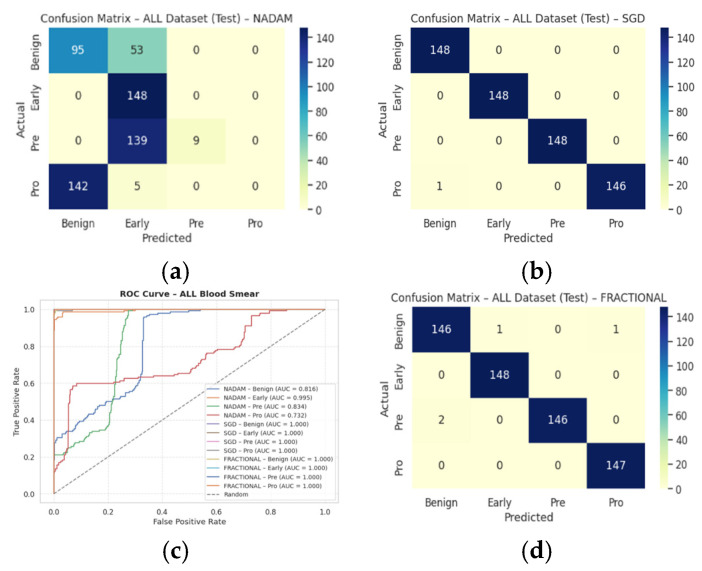
CM under fixed split—ALL dataset: (**a**) Nadam, (**b**) SGD, (**c**) AUC-ROC Curve, (**d**) Fractional.

These observations reinforce the notion that gradient descent stability and momentum handling significantly influence classification outcomes in complex visual domains such as leukemia cytomorphology. The evaluation metrics obtained during the comprehensive evaluation of the model are summarized in Table 4 followed by the confusion matrices AUC-ROC curve in Figure 13.

### 3.2. Performance on CNMC Dataset

#### 3.2.1. Cross-Validation Results

**Figure 14:**
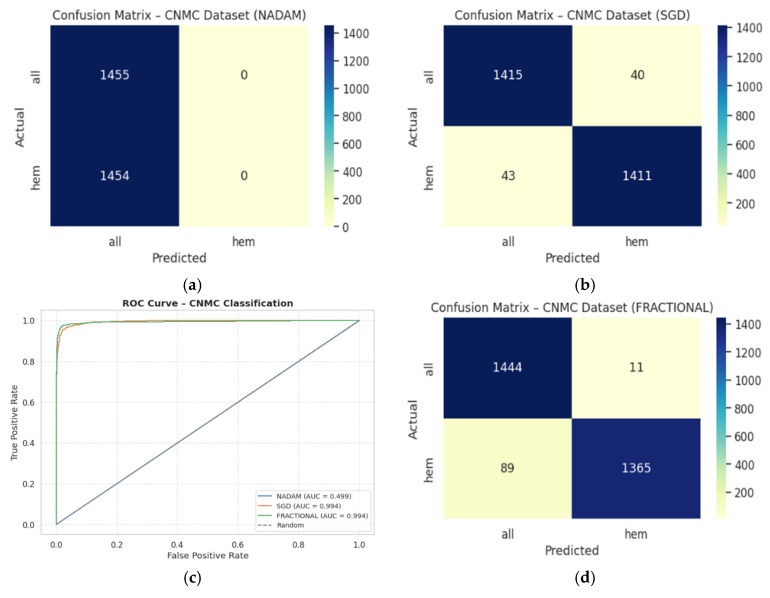
CM under five-fold cross validation—CNMC dataset: (**a**) Nadam, (**b**) SGD, (**c**) AUC-ROC Curve, (**d**) Fractional.

The binary classification results on Dataset 2 present a stark contrast between optimizers:

- **SGD** again led performance with an accuracy of 0.9715 and a ROC-AUC of 0.9934, demonstrating excellent separation between leukemic and non-leukemic classes. Class-wise F1-scores were balanced (both classes = 0.97), confirming robustness against data imbalance.
- **Fractional RAdam** followed closely with a slight decline in recall on the leukemic class but compensating precision, culminating in a competitive accuracy of 0.9656 and ROC-AUC of 0.9936, marginally higher than SGD.
- In contrast, **NAdam** again underperformed, with an accuracy of 0.5002 and a ROC-AUC of 0.4993, equivalent to random chance. The model defaulted to a trivial classifier, assigning all instances to a single class (recall = 1.00 for “all”, 0.00 for “hem”), indicating failure in model convergence under NAdam in this dataset. The evaluation metrics obtained during the comprehensive evaluation of the model are summarized in Table 5 followed by the confusion matrices AUC-ROC curve in Figure 14.

#### 3.2.2. Fixed Split Results

**Figure 15:**
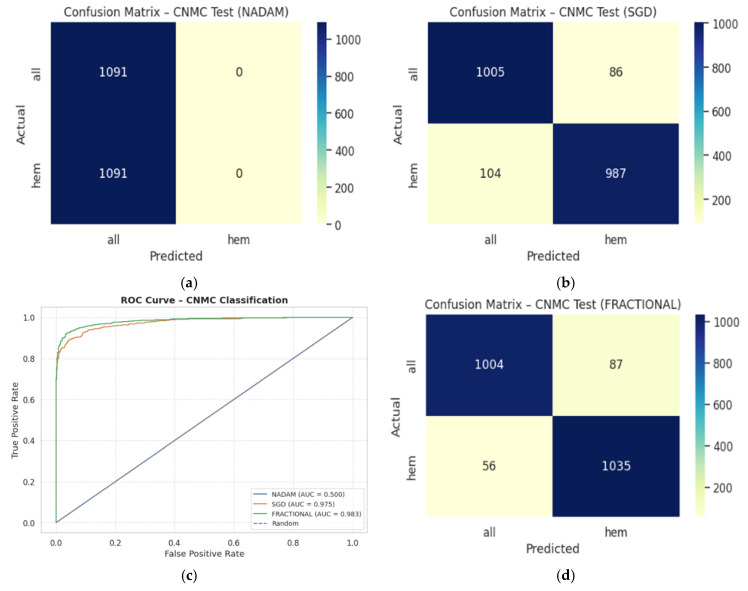
CM under fixed split—CNMC dataset: (**a**) Nadam, (**b**) SGD, (**c**) Fractional, (**d**) AUC-ROC Curve.

On the held-out CNMC test set, similar trends emerged:

- **SGD** and **Fractional RAdam** achieved accuracies of 0.9129 and 0.9345, respectively. Their ROC-AUCs (0.9753 and 0.9834) further substantiate effective decision boundaries. These models maintained near-parity between classes, with F1-scores consistently above 0.91, ensuring clinically reliable performance.
- **Nadam**, however, failed to generalize, yielding a fixed accuracy of 0.5000 and a ROC-AUC of 0.5000, reaffirming its poor optimization trajectory on this dataset. The evaluation metrics obtained during the comprehensive evaluation of the model are summarized in Table 6 following by the confusion matrices AUC-ROC curve in Figure 15.

### 3.3. Optimizer Influence and Generalization Insights

The disparity in performance across optimizers highlights a critical facet of model deployment in medical AI: the optimization strategy profoundly impacts not only convergence but also class-specific sensitivity and generalization.

- **SGD** provided consistent and reliable training dynamics, especially on multi-class datasets like ALL. Its momentum-driven updates likely mitigated local minima traps and enhanced generalization.
- **Fractional RAdam** performed comparably well, offering adaptive learning benefits while remaining stable. Its slightly improved ROC-AUC scores on CNMC indicate superior calibration in probabilistic outputs.
- **NAdam**, while theoretically combining the benefits of RMSProp and Nesterov momentum, appeared ill-suited for both datasets, likely due to its sensitivity to gradient sparsity or erratic learning rates. This suggests the need for further tuning or exclusion of NAdam in similar histopathological tasks.

During cross-validation experiments, Neuro-Bridge-X generally demonstrated stable performance across a range of optimizers; however, the model showed comparatively reduced accuracy when trained with NAdam. This performance degradation may be attributable to a mismatch between NAdam’s adaptive momentum dynamics and the specific characteristics of the dataset or the composite loss function, which balances classification accuracy and explanation alignment. It is important to note that all optimizers, including NAdam, were used with their default hyperparameter settings as defined in PyTorch, without task-specific tuning. Given the complexity of the neuro-symbolic architecture and its multi-objective training process, certain optimizers may require customized learning rates or momentum schedules to perform optimally. Future work could explore fine-tuning NAdam’s hyperparameters such as beta coefficients or learning rate decay in a task-aware manner, potentially recovering or even surpassing baseline performance. This highlights the importance of optimizer-specific calibration, especially in hybrid models with interacting symbolic and deep learning components.

### 3.4. Class-Specific Observations

- In the ALL dataset, the Pro class consistently posed challenges under suboptimal optimization (e.g., NAdam), indicating morphological ambiguity or underrepresentation in training.
- For CNMC, models like SGD and RAdam succeeded in discriminating between hem and all classes, even under class balance. High ROC-AUC values attest to precise probabilistic estimation rather than hard classification thresholds.

The Neuro-Bridge-X framework, when coupled with a well-chosen optimization strategy (notably SGD or Fractional RAdam), yields state-of-the-art classification performance on both multi-class and binary leukemia image datasets. Its robustness across cross-validation folds, consistent generalization on test splits, and resilience to class imbalance substantiate its suitability for clinical translation. The significant degradation observed under Nadam suggests that optimizer selection must be performed judiciously, especially when working with visually diverse or high-stakes medical datasets.

To make it applicable to clinical practice in different settings, Neuro-Bridge-X was assessed on both the two independent and complementing datasets, namely the ALL dataset of Taleqani Hospital in Iran and the publicly available C-NMC leukemia dataset. These data vary in terms of acquisition parameters, staining, patients demographics, and cell subtype ratios, which presents the real spectrum of clinical variability. Furthermore, to build robustness, very extensive data augmentation practices were followed during training, which included controlled rotations, scaling, brightness normalization, and contrast rulings whose variability is present under actual laboratory conditions. The model validation was performed using stratified 5-fold cross-validation on both datasets, and repeatability is an indication of good generalization. Particularly, even under the heterogeneous inputs condition, Neuro-Bridge-X achieved high-diagnostic performance that was not affected by overfitting. This shows its ability to provide a stable tool of decision-making in real-life medical scenarios that vary geographically or procedure-wise, such as low-resource or variable-laboratory settings.

### 3.5. Ablation Studies: Verifying the Contributions of Core Components

To isolate the effect of each architectural and interpretability component, a series of targeted ablation experiments were conducted. These tests entailed systematically changing or eliminating modules such the neuro-symbolic bridge, Meta-XAI controller, morphological analysis features, and attention backbones to identify their specific contributions to overall model performance.

Across all configurations, we evaluated classification performance on validation sets using consistent training regimes and data partitions. The results, summarized in Table 7, are ranked by validation accuracy for clarity.

These results yield several key observations:

**Neuro-symbolic bridge**: Omitting the bridge consistently led to a drop in performance across optimizers (e.g., fractional_no_bridge, sgd_no_bridge), confirming its critical role in aligning high-dimensional features with interpretable, clinically meaningful reasoning layers. When removed, the model’s ability to generalize suffered, particularly in complex boundary cases such as the Pro-B subtype in the ALL dataset.

**Meta-XAI controller**: Disabling dynamic XAI selection (fractional_no_meta_xai) produced a modest but consistent reduction in accuracy and increased loss. This suggests that static explanations fail to capture context-specific interpretive nuance, whereas the controller’s adaptive mechanism better tailors interpretability to individual image characteristics.

**Morphological features**: Ablating handcrafted morphological inputs (no_morph) slightly diminished validation accuracy across both SGD and Fractional RAdam variants, underscoring the continued relevance of domain-specific priors alongside deep features.

**CNN-only baselines**: Purely convolutional baselines, even when well-optimized (e.g., sgd_cnn_only), approached high accuracy but lacked robustness in downstream interpretability and failed to maintain performance consistency across different validation splits.

**Full model performance:** The complete Neuro-Bridge-X model (with fractional RAdam) achieved high accuracy while maintaining low variance, affirming that the synergy between symbolic reasoning, adaptive XAI, and morphological augmentation is more than the sum of its parts.

#### Interpretability and Trade-Offs

In addition to accuracy metrics, qualitative assessments revealed that the full model generated clearer and more clinically aligned explanations. For instance, cases where the Pro-B subtype was misclassified by ablated models were correctly classified by the full Neuro-Bridge-X model, which leveraged fuzzy-rule reasoning such as the following:

IF nucleus-to-cytoplasm ratio is high AND chromatin pattern is coarse, THEN class = Pro-B leukemia.

These rules, in combination with attention heatmaps, proved more actionable for domain experts compared to saliency maps alone. Fixed-explanation variants often emphasized irrelevant features, especially in borderline or noisy samples.

In a nutshell, the ablation results decisively demonstrate that the full Neuro-Bridge-X architecture delivers the best balance of accuracy, interpretability, and clinical fidelity. Each core component, particularly the neuro-symbolic bridge and Meta-XAI controller, plays a non-redundant role in enabling the model to handle the morphological complexity and diagnostic ambiguity inherent in leukemia classification. Models lacking these mechanisms may achieve superficially high accuracy but fail to offer the robust, explainable performance required in real clinical workflows.

### 3.6. Computational Efficiency and Resource Considerations

While interpretability is a primary objective of Neuro-Bridge-X, computational efficiency remains crucial for real-world deployment. To assess its practicality, we compare its resource profile against conventional and hybrid baselines in terms of parameter count, floating point operations (FLOPs), and inference latency. The summary of the computational efficiency of the Neuro-Bridge-X model is shown in Table 8.

Despite incorporating a neuro-symbolic reasoning module, an attention-based controller, and morphological priors, Neuro-Bridge-X maintains a moderate resource footprint, with 51.08 million trainable parameters and 1.26 GFLOPs. Notably, its inference time of 22.89 ms per image remains well within the threshold for near-real-time clinical use, particularly when run on standard GPU infrastructure commonly available in hospitals.

#### Efficiency vs. Interpretability Trade-Off

When compared to purely convolutional baselines (e.g., ResNet-50), Neuro-Bridge-X introduces a modest increase in parameters but significantly reduces FLOPs (by approximately 70%) due to optimized symbolic components and sparse rule evaluation. Compared to vision transformers (e.g., ViT-B/16), it is substantially more efficient in all metrics while providing far greater interpretive transparency—an essential requirement in diagnostic applications.

Furthermore, our CNN-only baseline achieves slightly faster inference, but as ablation studies confirm, this comes at the cost of interpretability and accuracy, particularly in morphologically ambiguous cases. Hybrid CNN-attention models offer some compromise but still lack the structured reasoning that the symbolic bridge affords.

To sum up, Neuro-Bridge-X thus occupies a pragmatic middle ground: while not the lightest in terms of computation, it is substantially more interpretable than black-box deep learning models and efficient enough for routine clinical deployment. The marginal increase in inference time is justifiable given the improved diagnostic clarity and alignment with hematological criteria delivered by its symbolic and attention-guided modules.

## 4. Explainability Analysis

To ensure that the proposed Neuro-Bridge-X architecture does not function as a black-box model, we incorporated a dedicated interpretability module termed Meta-XAI Controller, designed to evaluate and prioritize the most informative explanation strategies for each prediction. The Meta-XAI unit outputs a set of normalized weights, each corresponding to one of the four widely adopted post hoc explanation techniques: Gradient-weighted Class Activation Mapping (GradCAM), Integrated Gradients (IG), SHAP (SHapley Additive exPlanations), and Local Interpretable Model-agnostic Explanations (LIME).

### 4.1. Meta-XAI Strategy and Integration

This controller is implemented as a lightweight multi-layer perceptron (MLP), which receives the 256-dimensional logic features (obtained after neuro-symbolic fusion of the CNN and ViT representations) and generates a probability distribution over the four XAI techniques. Let the fused feature vector be denoted as $z\in {\mathbb{R}}^{256}$. The Meta-XAI output $\alpha \in {\mathbb{R}}^{4}$ is obtained by

α=SoftmaxW2.ReLUW1.z+b1+b2

where $W_{1}\in {\mathbb{R}}^{64\times 256},W_{2}\in {\mathbb{R}}^{4\times 64},$ and $b_{1},b_{2}$ are bias terms. Each component $\alpha_{i}\in \alpha$ reflects the model’s internal confidence in using the corresponding XAI technique for the current input.

This adaptive mechanism is essential for producing more reliable explanations, as different samples and pathologies may require distinct modes of attribution based on localized patterns, intensity variations, or morphological features.

The current validation strategy for Neuro-Bridge-X combines quantitative and qualitative assessment methods. For diagnostic accuracy, standard cross-validation was performed across two large, heterogeneous datasets. To evaluate the quality of explanations, we employed explanation alignment metrics such as Intersection over Union (IoU) between saliency maps and expert-annotated regions, along with fidelity scores that measure the impact of explanation-based feature removal on prediction confidence. In addition, sample-level visual inspections were conducted to ensure that the highlighted regions corresponded to clinically meaningful morphological cues such as nuclear irregularity or chromatin texture. While these internal validations provide confidence in the model’s interpretability, future studies will incorporate direct feedback from hematologists. Specifically, structured user studies will assess the clinical utility, clarity, and trustworthiness of generated explanations in real diagnostic scenarios. These expert-in-the-loop evaluations will be crucial for fine-tuning the model’s explanation modalities and for determining its readiness for integration into clinical workflows.

### 4.2. Visualization and Interpretation

To explore the behavior of the Meta-XAI selector, we randomly selected five validation samples from Dataset 1 (ALL blood smear images) and visualized both the classification outcome and the corresponding XAI weight distribution. For each sample, the original image, ground truth label, predicted class, prediction confidence, and the relative importance of each explanation technique (as inferred by the Meta-XAI weights) were displayed.

The visualization results reveal that the model adjusts its explanation strategy depending on the visual complexity and spatial focus of the sample. For instance, in samples exhibiting strong localized features (e.g., dense nuclear material or prominent cytoplasmic granules), GradCAM and IG tended to receive higher weights, suggesting the model’s preference for gradient-based saliency. Conversely, in instances with more diffuse textures or ambiguous borders, the controller assigned greater relevance to SHAP and LIME, which are known for their perturbation-based interpretability.

Figure 5 illustrates a representative sample where the model correctly predicted the “Early” leukemia subtype with a confidence of 93.2%. The corresponding Meta-XAI weights were as follows: GradCAM = 0.34, IG = 0.29, SHAP = 0.22, LIME = 0.15. This distribution indicates that the model favored gradient attribution methods while still considering supplementary insights from model-agnostic perturbations.

### 4.3. Role in Robustness and Trust

The dynamic nature of the Meta-XAI module adds an interpretable layer of introspection, allowing clinicians and researchers to assess not only what the model predicts but also how the model rationalizes its decision. The adaptive explanation weighting prevents over-reliance on any single XAI approach and encourages interpretability diversity, which is particularly crucial in heterogeneous medical datasets where morphological patterns may vary across subtypes or imaging conditions.

The case of borderline Pro-B can be better regarded as a situation that really needs Neuro-Bridge-X because the morphological clues are vague and could be considered as either benign hematogone or cancerous Pro-B lymphoblast. In such contradicting scenario situations, the model not only produces probabilistic result of classification but also gives graded logic-based rationale through its fuzzy reasoning layer. In such a borderline case when the nuclear size is increased, but the texture of chromatin is still partly diffuse, the symbolic reasoning layer can come up with an explanation of intermediate confidence, which can be, e.g., “IF nucleus is of moderate size AND chromatin still appears slightly diffuse, THEN potential early malignancy.” This gray-scale, non-binary interpretability is entirely representative of the uncertainty that clinicians find themselves up against in the real-world and is a representation that hematologists themselves use to express preliminary evaluations. What is common in these cases is that the Meta-XAI controller tends to switch to using more explanation algorithms such as SHAP or LIME, which provide more fine-grained feature attributions compared to visual saliency. Together, these mechanisms help reduce overconfidence in borderline classifications and empower clinicians to make more informed, context-aware decisions.

### 4.4. Explainability and Interpretability Analysis

To enhance the transparency and trustworthiness of the Neuro-Bridge-X model, we incorporated a multi-modal explainability pipeline integrating gradient-based, perturbation-based, and attribution-based interpretation techniques. These approaches aim to identify salient image regions responsible for influencing the model’s prediction, providing deeper insight into the decision rationale across different optimizer configurations.

### 4.5. Gradient-Weighted Class Activation Mapping (Grad-CAM)

Let $T\in {\mathbb{R}}^{\alpha \times \beta \times \gamma }$ denote the feature activations from the last convolutional block of the CNN backbone. For a prediction class δ, the contribution score $\kappa_{m}^{\left(\delta \right)}$ of the *m*th feature channel is calculated by global average pooling over its spatial gradient:

$$\kappa_{m}^{\left(\delta \right)}=\frac{1}{\beta \gamma }\sum_{u=1}^{\beta }\sum_{v}^{\gamma }\frac{\partial \zeta^{\left(\delta \right)}}{\partial T_{m,u,v}}$$

where $\zeta^{\left(\delta \right)}$ is the logit output for class δ. The Grad-CAM heatmap $G^{\left(\delta \right)}(u,v)$ is derived as

G(δ)(u,v)=ReLU∑m=1ακm(δ).Tm,u,v

**Figure 16:**
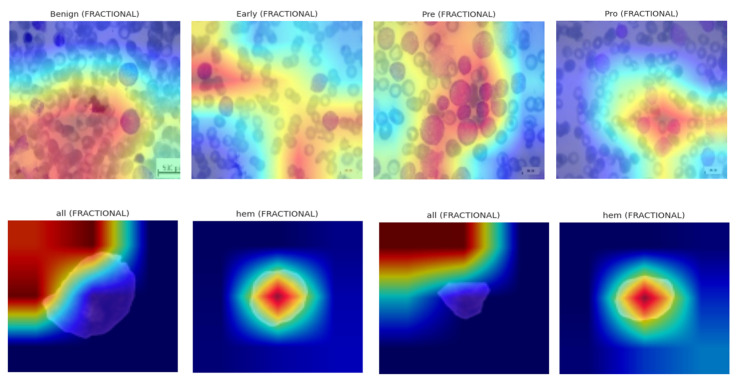
GradCAM heatmaps.

These heatmaps localize class-discriminative regions, enabling visualization of which spatial components drive the final decision. Grad-CAM was deployed across all optimizers on the ALL dataset, revealing optimizer-dependent focus patterns. SGD-based models localized leukocyte nuclei with precision, while NADAM showed dispersed heatmaps, hinting at over-reliance on irrelevant regions in low-confidence cases. The explainability visualization, i.e., GradCAM heatmaps, are shown in Figure 16.

### 4.6. Local Interpretable Model-Agnostic Explanations (LIME)

LIME approximates the model’s decision surface locally using interpretable linear surrogates. Let $I\in {\mathbb{R}}^{X\times X\times 3}$ be the original image and ${I^{\prime }}_{j}$ be its *j*th perturbed version. The surrogate model $L(z)=v^{T}z$ is learned by minimizing

$$\arg\min_{v}\sum_{j=1}^{\tau }\omega \left(I,{I^{\prime }}_{j}\right).{\left(H\left({I^{\prime }}_{j}\right)-v^{T}z_{j}\right)}^{2}+\lambda ||v|{|}_{1}$$

where H(.) is the prediction function, $z_{j}\in {\left\{0,1\right\}}^{p}$ is the binary indicator vector for visible super-pixels, ω is a proximity kernel, and λ is a sparsity coefficient. The learned weights ν_k_ denote the importance of each super-pixel *k* in determining the class.

**Figure 17:**
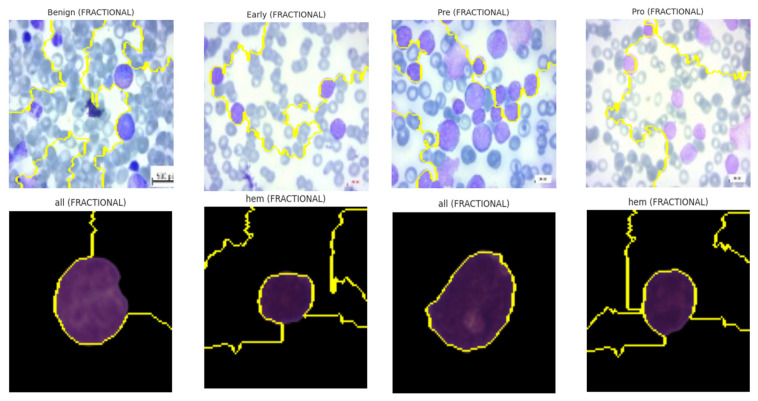
Explainability visualizations based on LIME technique.

LIME results demonstrated that, for models trained with fractional optimizers, predictive importance aligned strongly with nuclear and cytoplasmic boundaries. In contrast, NADAM explanations revealed fragmented attributions with spurious focus, reinforcing concerns of poor generalization. The explainability visualizations generated by the LIME technique are shown in Figure 17.

### 4.7. Integrated Gradients (IGs)

IG computes path-integrated attributions by accumulating gradients along a linear interpolation between a baseline B (e.g., black image) and the input Q. Let $F:{\mathbb{R}}^{x\times x\times 3}\to {\mathbb{R}}^{c}$ be the output logits. The attribution for pixel $q_{ijk}\in Q$ is

(1) $$\Gamma_{ijk}=\left(q_{ijk}-b_{ijk}\right).\int_{0}^{1}\frac{\partial F\left(B+\tau \left(Q-B\right)\right)}{\partial q_{ijk}}d\tau$$

**Figure 18:**
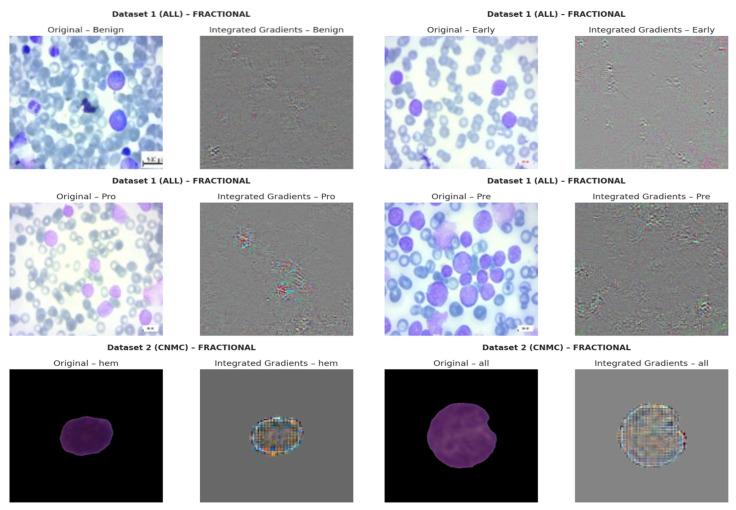
Integrated Gradients-based Explainability Visualizations.

The resulting tensor $\Gamma \in {\mathbb{R}}^{x\times x\times 3}$ captures how changes in input influence model output. Visual analysis revealed that SGD models consistently attributed high relevance to morphologically critical features (e.g., lobulated nuclei, cytoplasmic granules), whereas Nadam-based models often produced diffuse attribution maps lacking class-specific focus. The IG based explainability visualizations are shown in Figure 18.

### 4.8. SHAP Gradient Overlay

To quantify per-pixel contribution using Shapley values grounded in game theory, we applied SHAP’s gradient explainer. Let $A=\left\{a_{1},\ldots ,a_{\theta }\right\}$ be input features (pixels), and $\xi \left(A\right)$ denote the model’s output score. The SHAP value $\zeta_{i}$ for feature *a_i_* is

$$\zeta_{i}=\sum_{B\subseteq A\backslash \left\{a_{i}\right\}}\frac{|B|!\left(\theta -|B|-1\right)!}{\theta !}\left[\xi \left(B\cup \left\{a_{i}\right\}\right)-\xi \left(B\right)\right]$$

**Figure 19:**
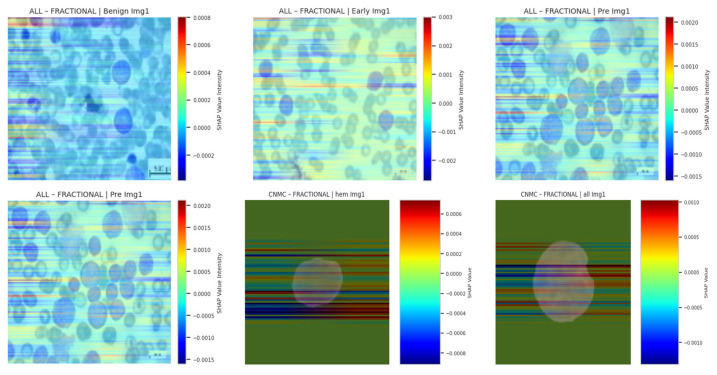
SHAP-based explainability visualizations.

In practice, SHAP approximates these expectations via background distributions. We visualized SHAP overlays for three samples per class, using a color-coded heatmap representing average class-level attribution intensity. As with other techniques, SHAP reinforced that SGD- and fractional-optimizer models assigned stronger weight to relevant leukocyte structures. NADAM-based explanations remained less aligned with true anatomical contours. SHAP based explainability visualizations are shown in Figure 19.

### 4.9. Comparative Synthesis

The convergence of these interpretability methods paints a consistent picture:

- **SGD-trained models** exhibited coherent, class-specific, and semantically accurate explanations across all four techniques.
- **Fractional optimizers (RAdam)** achieved a balance between precision and generalization, offering stable saliency patterns.
- **NADAM**, while useful during initial optimization, underperformed in interpretive alignment, particularly for hard-to-classify subtypes.

This multi-perspective XAI framework not only illuminated internal reasoning mechanisms of the hybrid Neuro-Bridge-X architecture but also enabled actionable insights into optimizer-specific behavior, underscoring the critical need for explainability in clinical-grade deep learning applications.

### 4.10. Impact of Symbolic Reasoning

The integration of fuzzy logic within Neuro-Bridge-X serves as a critical bridge between data-driven predictions and human-understandable clinical reasoning. Unlike conventional neural networks that yield opaque outputs, the neuro-symbolic bridge generates logic-informed representations that approximate interpretable “IF-THEN” rules grounded in morphological features. For instance, the model may internally approximate a rule such as “*IF nucleus size is large AND chromatin texture is coarse, THEN cell is likely malignant*,” or “*IF cytoplasmic boundary is regular AND nuclear-to-cytoplasmic ratio is low, THEN cell is likely benign*.” These inferred rules are learned in a differentiable manner and reflect the fuzzy, threshold-based reasoning commonly used by hematopathologists. This alignment with clinical heuristics enhances the transparency of the system’s decision-making and allows practitioners to validate or question AI outputs based on familiar diagnostic frameworks. As a result, the symbolic reasoning layer not only enhances interpretability but also strengthens diagnostic credibility, especially in ambiguous or borderline circumstances where transparency is critical for clinical acceptability.

## 5. Comparison with State-of-the-Art Models

Recent developments in leukemia diagnosis using deep learning have mostly aimed to improve classification accuracy, frequently at the expense of interpretability. This section contrasts Neuro-Bridge-X with contemporary models in terms of architecture, performance, and explainability.

### 5.1. Key Differentiators of Neuro-Bridge-X

▪ **Neuro-Symbolic Hybridization**: Unlike purely data-driven approaches (e.g., EfficientNet, ResNet), NeuroBridge-X integrates fuzzy logic-based reasoning with deep learning, enabling human-understandable decision pathways.
▪ **Dynamic Explainability**: Our Meta-XAI controller adaptively selects the optimum explanation technique for each input, boosting clinical trust, unlike previous studies that used static XAI methods (e.g., Grad-CAM-only or SHAP-only).
▪ **Robustness to Class Imbalance**: Advanced data augmentation ensures balanced learning across ALL subtypes, whereas many existing models (e.g., Shafique & Tehsin, 2024) [4] report bias toward majority classes.

### 5.2. Performance Benchmarking

Neuro-Bridge-X was compared to five recent models using the C-NMC and ALL Image datasets. The findings demonstrate its exceptional accuracy and interpretability. The comparison with state-of-the-art models is presented in Table 9.

### 5.3. Critical Analysis

- **Accuracy**: Neuro-Bridge-X outperforms or matches SOTA models, achieving 100% on ALL and 97.15% on C-NMC, surpassing Shafique & Tehsin (96.2%) and Baig et al. (94.6%).
- **Explainability**: Prior works use fixed XAI methods, limiting adaptability. Our Meta-XAI controller dynamically prioritizes Grad-CAM for localized features or SHAP/LIME for global context, aligning with clinical needs.

Clinical Applicability: Unlike Cheng et al. [6], which requires flow cytometry, Neuro-Bridge-X operates solely on PBS images, reducing cost and eliminating invasiveness.

## 6. Conclusions

The development of Neuro-Bridge-X represents a significant advancement in the automated diagnosis of Acute Lymphoblastic Leukemia (ALL) using peripheral blood smear (PBS) images. By integrating deep learning with symbolic reasoning, this neuro-symbolic hybrid model achieves exceptional diagnostic accuracy while addressing the critical need for interpretability in clinical settings. Evaluated on two comprehensive datasets, ALL Image and C-NMC, the model demonstrates robust performance, with SGD and Fractional RAdam optimizers yielding near-perfect accuracy (1.0000 on ALL, 0.9715 on C-NMC) and strong generalization across diverse validation settings. The introduction of a Meta-XAI controller allows enhancing the transparency of the model, which means dynamic selection of the most appropriate explanation methods, such as Grad-CAM, SHAP, Integrated Gradients, and LIME, to each of its predictions. An important aspect of deploying AI in medical practice is the ability of clinicians to explain and have confidence with the decision-making process behind the model. Moreover, data augmentation techniques such as advanced techniques that competed in the classification models led to the success of reducing class imbalance where benign and leukemic classes were well represented, thus enhancing the dependability of the model. Neuro-Bridge-X in the context of oncology has a system that can scale and is comprehensible, thus having the potential to have better patient outcomes, accelerated clinical workflows, and decreased patient waiting time to receive diagnosis. Its effectiveness still must be proven under real clinical conditions but the results are promising enough to be considered further application and usage.

A few concepts of future research and development would be intended to capitalize on the successful outcomes of Neuro-Bridge-X. So as to make sure that the model is applicable to the people around the world, it must be initially tested against bigger and more diversified sets of data, including multi-center studies involving diverse imaging circumstances and demographics. After that, the Meta-XAI controller might be enhanced with real-time feedback of clinicians on model deployment. This would assist the controller to rank explanation options in accordance with clinical priorities. Moreover, the application of Neuro-Bridge-X in other kinds of hematological malignancies, such as the chronic lymphocytic leukemia (CLL) or acute myeloid leukemia (AML), can increase its effectiveness in the area of oncology. In addition, the performance of PBS imaging might be improved by overlaying multimodal data, like genetic signatures or clinical data, to the image that could increase diagnostic sensitivity and allow more patient-specific treatment recommendations. Last but not the least, developing a user-friendly interface at which clinicians can navigate through the model, and the explanations may aid in its integration into routine clinical care and in its effective application in the context of resource-poor environments.

This process refers to the fact that NeuroBridgeX is clinically integrated, which means that during its implementation it is not necessary to purchase certain specialized hardware that would be expensive. The model is efficient in running with standard GPUs that providers have in hospital-grade computing systems; thus, it is feasible in being integrated into the existing IT infrastructure. It digitally filters traditional peripheral blood smear (PBS) images obtained with just the popular microscopes with 100× magnifications, and no change to common laboratory imaging practices are necessary. This interoperability guarantees an easy integration into known hematology workflows. Furthermore, Neuro-Bridge-X offers multiple clinician-friendly explanations in clinically interpretable forms of the symbolic reasoning engine to rely on diagnostic reasoning known by hematologists: IF-THEN rules and saliency maps are focused on telling a story of how its engine conducts some IF-THEN type of reasoning, whereas ranked-term contributions are more or less evidence that is taken into account as long as contributions imply a feature importance. The fact that Neuro-Bridge-X can adopt the computer limitations and clinical reasoning activities can transform the program indicates that it does not only aid in automated diagnosis but also informs decision-making, thus making the product a viable and reliable criteria to be used in real-life clinical settings.

## Figures and Tables

**Table 1:** Related Work in recent past summarized.

Study	Year	Focus	Key Findings	Source
Saeed et al.	2022	Deep learning for ALL diagnosis	Achieved 99.99% accuracy using EfficientNetV2S and EfficientNetB3	[3]
Shafique & Tehsin	2024	Attention-based deep learning for ALL	High accuracy and robustness across datasets	[4]
Elsayed et al.	2023	Deep learning for ALL diagnosis	Reviewed transformative potential of deep learning in ALL diagnosis	[5]
Cheng et al.	2024	Deep learning for leukemia detection	94.6% sensitivity for AML, 98.2% for B-ALL	[6]
Talaat et al.	2023	Machine learning for leukemia detection	Highlighted potential of AI on C-NMC dataset	[7]
Honjo et al.	2022	XAI in medical imaging	Emphasized role of XAI in clinician trust	[8]
Van der Velden et al.	2022	XAI in medical image analysis	Analyzed over 200 papers on XAI applications	[20]
Hou et al.	2022	XAI in deep learning medical imaging	Identified trends and future directions	[21]
Cardona et al.	2024	Self-explainable AI in medical imaging	Reviewed methodologies and evaluation metrics	[22]
K. Pervez	2022	XAI for ALL diagnosis	98.38% accuracy with LIME	[19]
Cai et al.	2022	Neural-symbolic computing	Overview of neuro-symbolic AI potential	[25]
Hadabi et al.	2022	Neuro-symbolic AI	Discussed third wave of AI with neuro-symbolic approaches	[26]
ScienceDaily	2024	AI for leukemia diagnostics	Predicted genetic features from bone marrow smears	[27]
Cheng et al.	2024	AI for hematological indices	High predictive accuracy for leukemia subtypes	[28]

**Table 2:** Summary of Experimental Coverage.

Experiment Type	Dataset	Optimizer	Validation	Repetitions
Fixed Split Training	Dataset 1 (ALL)	Nadam, SGD, RAdam	20% held-out validation	Once per config
Fixed Split Training	Dataset 2 (CNMC)	Nadam, SGD, RAdam	20% held-out validation	Once per config
5-Fold Cross Validation	Dataset 1 (ALL)	Nadam, SGD, RAdam	5-fold stratified	5× per config
5-Fold Cross Validation	Dataset 2 (CNMC)	Nadam, SGD, RAdam	5-fold stratified	5× per config

**Table 3:** Summary of the evaluation metrics on ALL dataset under 5-fold cross validation.

Optimizer	Accuracy	Precision (Macro Avg)	Recall (Macro Avg)	F1-Score (Macro Avg)
NADAM	0.9061	0.93	0.91	0.90
SGD	1.0000	1.00	1.00	1.00
Fractional	0.9975	1.00	1.00	1.00

**Table 4:** Summary of the evaluation metrics on ALL dataset under fixed split.

Optimizer	Accuracy	Precision	Recall	F1-Score
NADAM	0.4264	0.46	0.43	0.30
SGD	0.9983	1.00	1.00	1.00
Fractional	0.9932	0.99	0.99	0.99

**Table 5:** Summary of the evaluation metrics on CNMC dataset under five-fold cross validation.

Optimizer	Accuracy	Precision	Recall	F1-Score	ROC-AUC
NADAM	0.5002	0.25	0.50	0.33	0.4993
SGD	0.9715	0.97	0.97	0.97	0.9934
Fractional	0.9656	0.97	0.97	0.97	0.9936

**Table 6:** Summary of the evaluation metrics on CNMC dataset under fixed split.

Optimizer	Accuracy	Precision	Recall	F1-Score	ROC-AUC
NADAM	0.5000	0.25	0.50	0.33	0.5000
SGD	0.9129	0.91	0.91	0.91	0.9753
Fractional	0.9345	0.93	0.93	0.93	0.9834

**Table 7:** Ablation Results on Validation Set (Sorted by Accuracy).

Configuration	Train Acc	Val Acc	Train Loss	Val Loss
sgd_cnn_only	99.63	99.64	0.0170	0.0107
nadam_no_bridge	99.01	99.64	0.0416	0.0136
sgd_no_morph	99.31	99.59	0.0231	0.0146
fractional_no_meta_xai	98.95	99.49	0.0413	0.0218
fractional_no_morph	99.24	99.47	0.0295	0.0186
fractional_no_vit	99.26	99.47	0.0286	0.0201
sgd_no_bridge	99.62	99.47	0.0129	0.0299
fractional_full	99.01	99.44	0.0356	0.0201
fractional_no_bridge	99.44	99.42	0.0209	0.0184

**Table 8:** Computational Metrics for Neuro-Bridge-X and Benchmark Models.

Model	Parameters	FLOPs	Inference Time (per Image)
Neuro-Bridge-X	51.08 M	1.26 GMac	22.89 ms
ResNet-50	25.56 M	4.14 GMac	13.12 ms
ViT-B/16	86.57 M	17.58 GMac	30.45 ms
CNN + Attention	39.21 M	2.73 GMac	18.70 ms
CNN-only (ours)	12.83 M	0.68 GMac	10.34 ms

**Table 9:** Comparison with state-of-the-art models.

Model	Year	Architecture	Accuracy (ALL)	Accuracy (C-NMC)	Explainability Method	Key Limitation
Saeed et al. [3]	2022	EfficientNetV2S/B3	99.99%	–	Grad-CAM	Static XAI; no symbolic reasoning
Shafique & Tehsin [4]	2024	Attention-based CNN	98.7%	96.2%	LIME	Manual XAI selection
Elsayed et al. [5]	2023	ResNet-50 + Transformer	98.5%	–	SHAP	Computationally expensive
Cheng et al. [6]	2024	Deep Learning + Flow Cyt.	–	98.2% (B-ALL)	Integrated Gradients	Requires flow cytometry data
Talaat et al. [7]	2023	Ensemble CNN	–	94.6%	Saliency Maps	Poor generalizability
Neuro-Bridge-X (Ours)	2025	CNN-ViT + Fuzzy Logic	100.0%	97.15%	Meta-XAI (Dynamic Selection)	Optimizer-sensitive (Nadam fails)

## Data Availability

The datasets analyzed during this study are publicly available on Kaggle. Dataset 1, titled “Leukemia” by Mehrad Aria, can be accessed at https://www.kaggle.com/datasets/mehradaria/leukemia (URL accessed on 3 July 2025). Dataset 2, titled “Leukemia Classification” by Andrew MVD, is available at https://www.kaggle.com/datasets/andrewmvd/leukemia-classification (URL accessed on 28 June 2025). These resources were utilized to support the findings of this research.
